# Non‐Anatomical Arthroscopic All‐Inside Repair of Medial Meniscus Posterior Root Tear to Posterior Cruciate Ligament for Patients with Normal Lower Limb Alignment

**DOI:** 10.1111/os.13222

**Published:** 2022-02-24

**Authors:** Jun Jiang, Dang Xing, Lei Ni, Jian Chen

**Affiliations:** ^1^ Arthritis Clinic & Research Center (ACRC) Peking University People Hospital Beijing China

**Keywords:** Medial meniscus posterior root tear, Non‐anatomical all‐inside arthroscopic repair, Normal lower limb alignment, Posterior cruciate ligament

## Abstract

**Objective:**

To describe a non‐anatomical arthroscopic all‐inside repair of medial meniscus posterior root tear (MMPRT) to posterior cruciate ligament (PCL) technique for patients with normal lower limb alignment and to evaluate the short‐term clinical and radiologic outcomes.

**Methods:**

MMPRT directly to PCL was repaired with all‐inside horizontal mattress suturing technique rather than by the transtibial pullout suture technique or anchor suturing repair technique in 20 Laparade Type II MMPRT patients with normal lower limb alignment during 2018–2019. The clinical and radiological outcomes were evaluated retrospectively for at least 2 years follow‐up. The VAS score, Lysholm score, Tegner activity score were evaluated preoperatively and at the final follow‐up. The status of the medial meniscus posterior root were assessed on magnetic resonance imaging (MRI) preoperatively and at the final follow‐up.

**Results:**

Twenty patients (mean age 54.5 ± 19.5 years) were included in the present study. The mean follow‐up duration was 32.5 ± 5.8 months. The VAS score was significantly decreased from preoperative 6.5 ± 1.5 to 2.1 ± 1.4 at the final follow‐up (*P* < 0.01). The mean Lysholm score was significantly improved from 43.7 ± 10.9 preoperatively to 85.7 ± 10.8 (*P* < 0.01). The median Tegner activity score was improved from 1.0 (range 1–4) to 3.0 (range 2–4, *P* < 0.01). On MRI, a total of 12 cases (60%) had complete healing, while eight cases (40%) had partial healing.

**Conclusion:**

Non‐anatomical arthroscopic all‐inside repair of MMPRT to PCL may yield beneficial clinical outcomes and a higher rate of clinical healing in Type II MMPRT patients with normal lower limb alignment. It is an easy and reliable alternative technique to the transtibial pullout suture or anchor suture repair technique.

## Graphical abstract

Non‐anatomical arthroscopic all‐inside horizontal mattress suturing repair of MMPRT to PCL in type II MMPRT patients with normal lower limb alignment yielded beneficial clinical outcomes and a higher rate of clinical healing. This technique could be an alternative to the transtibial pullout suture or anchor suture repair technique.

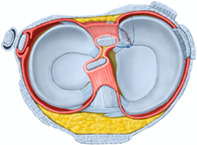

## Introduction

The medial meniscus posterior root (MMPR) provided pivotal role in maintaining the hoop tension of medial meniscus and native tibiofemoral contacting biomechanics. Medial meniscus posterior root tear (MMPRT) (radial tear up to 9–10 mm from the posterior root attachment) significantly altered the native biomechanics of the posterior meniscal roots^1^, ^2^. The prevalence of MMPRTs ranged from 10.1% to 21.4% in medial meniscus tears^3^, ^4^, ^5^ and with 3.6% of total meniscal tears^6^.

Sometimes subtotal meniscectomy had to be conducted due to biomechanical instability of untreated MMPRTs. But MMPR was important in maintaining normal knee cartilage^7^, ^8^. Medial meniscus posterior root (MMPR) acts as an anchor into posterior part of medial tibial plateau for medial meniscus, which lies between PCL tibial insertion and medial tibial eminence^9^. MMPR also has a ligament structure behind the PCL to connect the posterior knee septum. Thus, MMPR provides hoop tension for medial meniscus's function of shock absorption, load transmission, and knee stability. Posterior root tear of medial meniscus can cause knee osteoarthritis because of hoop tension loss and medial meniscal extrusion^10^, ^11^, ^12^. Therefore, it was important to repair MMPRT anatomically and to restore normal biomechanics of the MMPR, especially the anatomy and hoop tension of medial meniscus.

Anatomical repair of an MMPRT with transtibial pullout suture or anchor suture repair could restore both the anatomy, hoop tension and loading ability of medial meniscus^13^, ^14^, ^15^ and resulted in almost intact contact area and minimal increases in contact pressures compared with the intact knee^16^. However, this arthroscopic procedure was technically demanding and time‐consuming. Further, it could cause iatrogenic damage to native knee cartilage^17^. Saltzman *et al*. reported that non‐anatomical suture fixation repair of MMPRTs to posterior cruciate ligament (PCL) improved the contact area and resulted in almost same contact pressure as the intact knee through cadaver biomechanical study. The authors concluded that the non‐anatomical technique might be an alternative to traditional transtibial pullout suture or anchor suture repair^18^.

In 2018 we began to conduct non‐anatomical arthroscopic repair of MMPRT directly to PCL in Type II MMPRT patients with normal lower limb alignment. In the present study, we retrospectively reviewed the clinical and radiological outcomes of the non‐anatomical arthroscopic technique. The purpose of this study was: (i) to describe this non‐anatomic arthroscopic all‐inside repair technique for MMPRTs patients with normal lower limb alignment; and (ii) to evaluate the short‐term clinical outcome and radiologic healing results. Our hypothesis was that (i) non‐anatomical arthroscopic repair of Type II MMPRT directly to PCL could yield beneficial clinical outcome and a higher clinical healing rate; and (ii) the easy and reliable all‐inside arthroscopic repair method was an alternative method to arthroscopic tibial pullout suture or anchor suture repair for Type II MMPRT.

## Materials and Methods

**Fig. 1:**
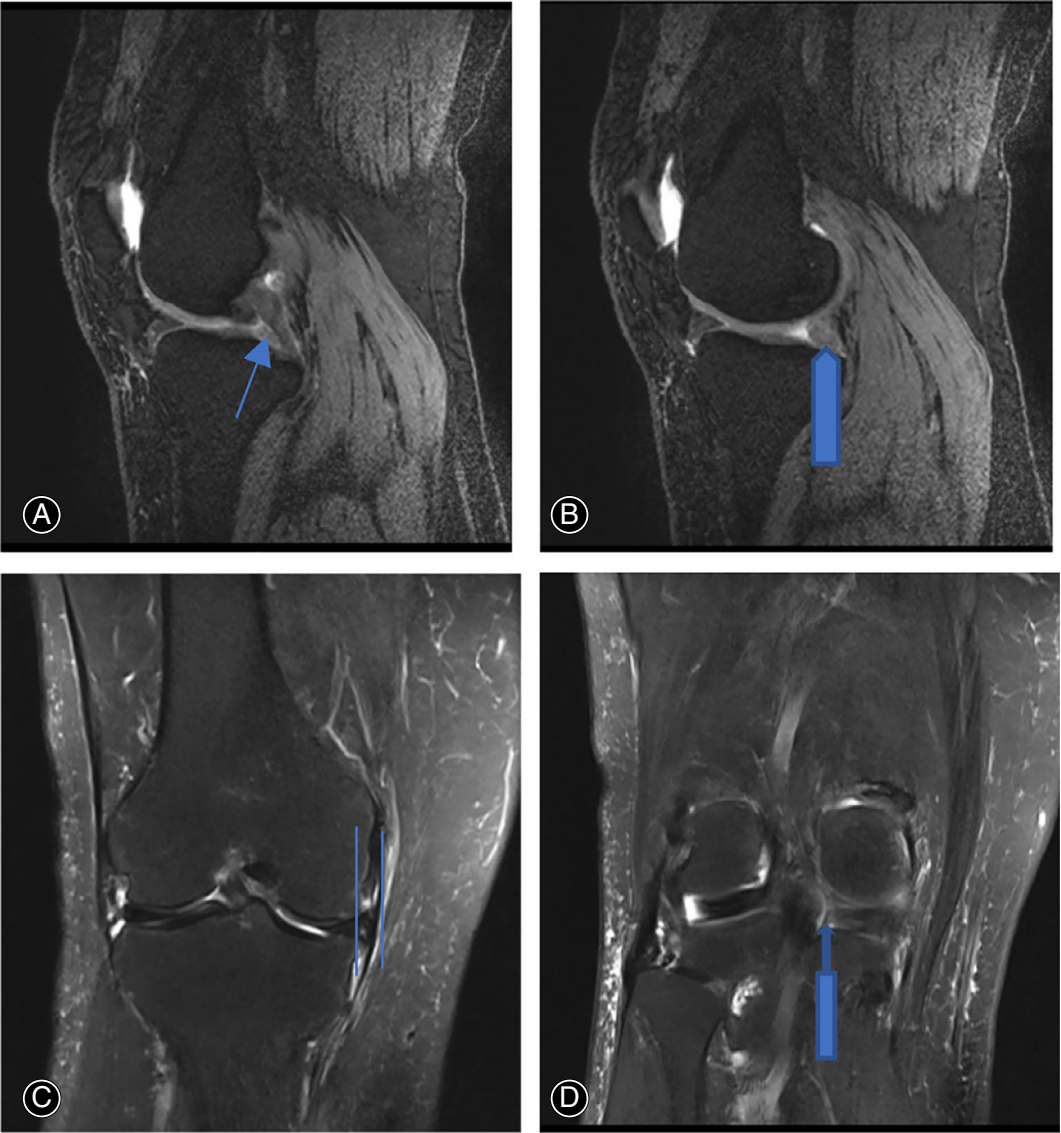
Preoperative MRI images of a 67‐year‐old woman patient with right knee MMPRT and normal limb alignment. (A) Ghost sign in T2 sagittal view indicated by long fine arrow (B) round shape (not as normal triangular shape) of medial meniscus posterior root in T2 sagittal view indicated by long coarse arrow (C) medial meniscus extrusion in T2 coronal view indicated by two lines (D) left sign in T2 coronal view indicated by long coarse upper arrow.

**Fig. 2:**
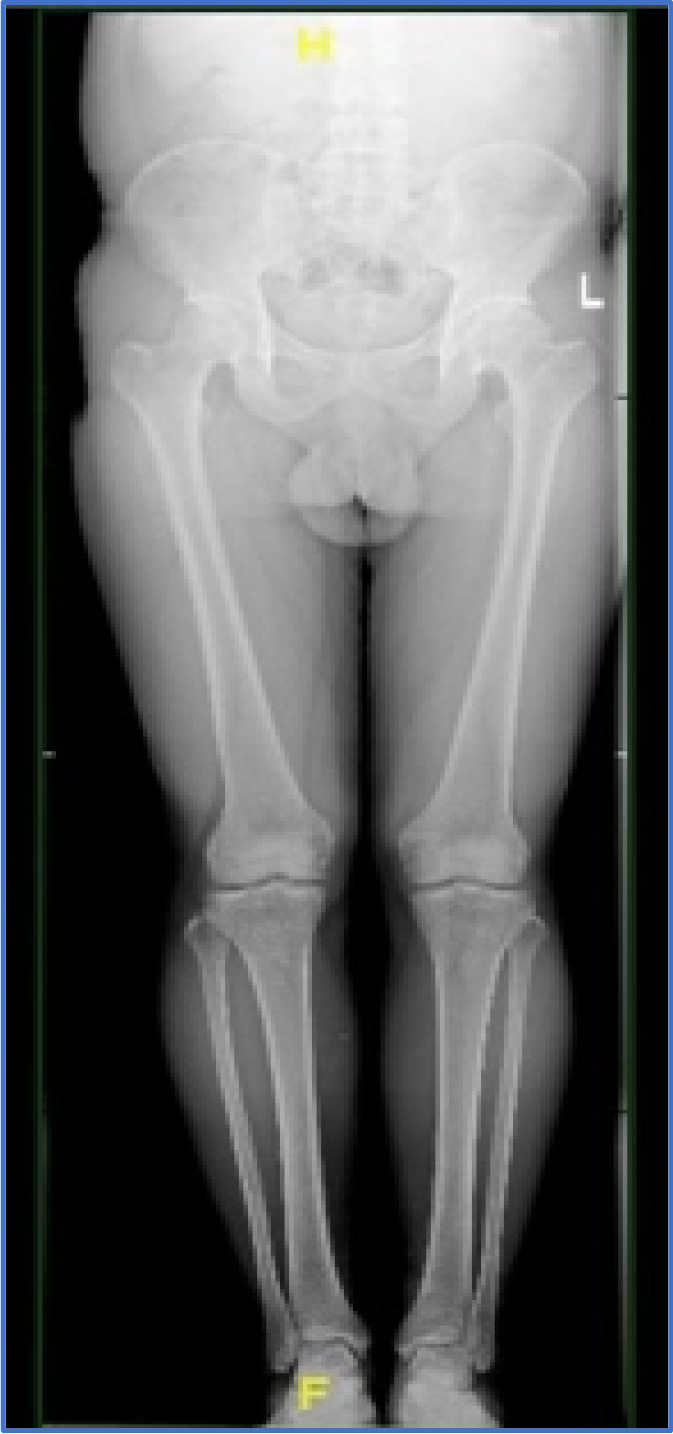
Normal mechanic alignment of right lower limb in standing full‐length radiograph.

This study was approved by the Institutional Review Board of Peking University People hospital before commencement. From January 2018 to August 2019, a total of 20 patients with type II MMPRTs (complete radial tears of posterior root) and normal lower limb alignment underwent arthroscopic all‐inside non‐anatomical suture fixation to PCL. Patients without obvious injury had a sudden posteromedial painful popping sensation and/or squatting limitation symptom and local tenderness sign. McMurray test was positive with deep knee flexion and tibial external rotation. Preoperative MRI was performed to confirm MMPRT with cleft sign and ghost sign (Figure 1). Weight‐bearing, full‐length AP radiograph of lower limb was performed to confirm normal mechanical alignment (Figure 2). The inclusion criteria included: (i) reparable Type II MMPRT was confirmed through arthroscopic examination; (ii) normal lower limb mechanical alignment; (iii) pre‐ and postoperative MRI were available; and (iv) clinical and radiological outcomes were available for at least 2 years follow‐up. The exclusion criteria included: (i) patients with knee varus deformity; (ii) MMPRT combined with ligament injury; and (iii) concomitant medial meniscus body or lateral meniscus tear.

### Surgical Techniques

**Fig. 3:**
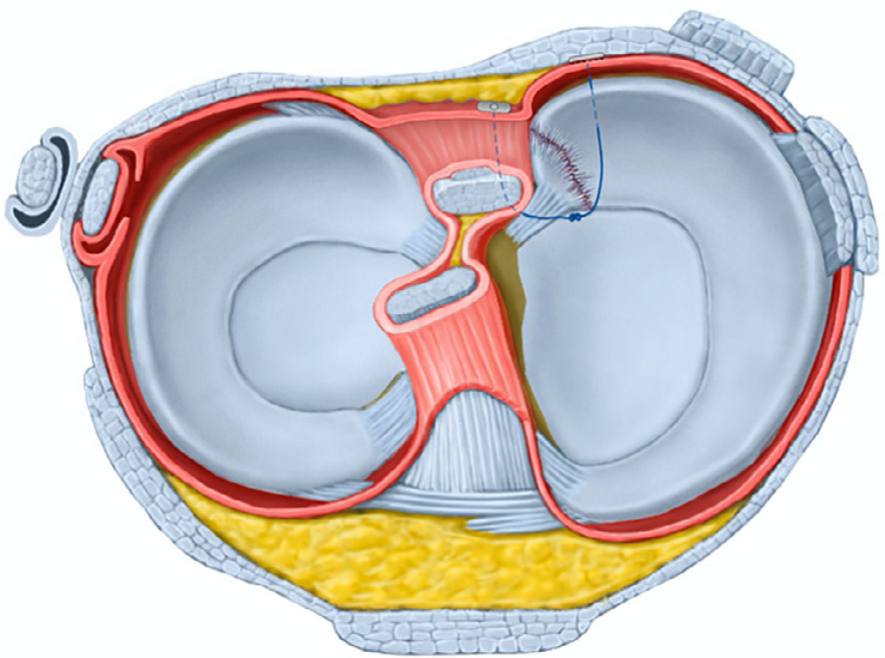
Schematic drawing of the horizontal mattress suturing configuration for MMPRT to PCL using FAST‐FIX 360° meniscal repair system sutures.

**Fig. 4:**
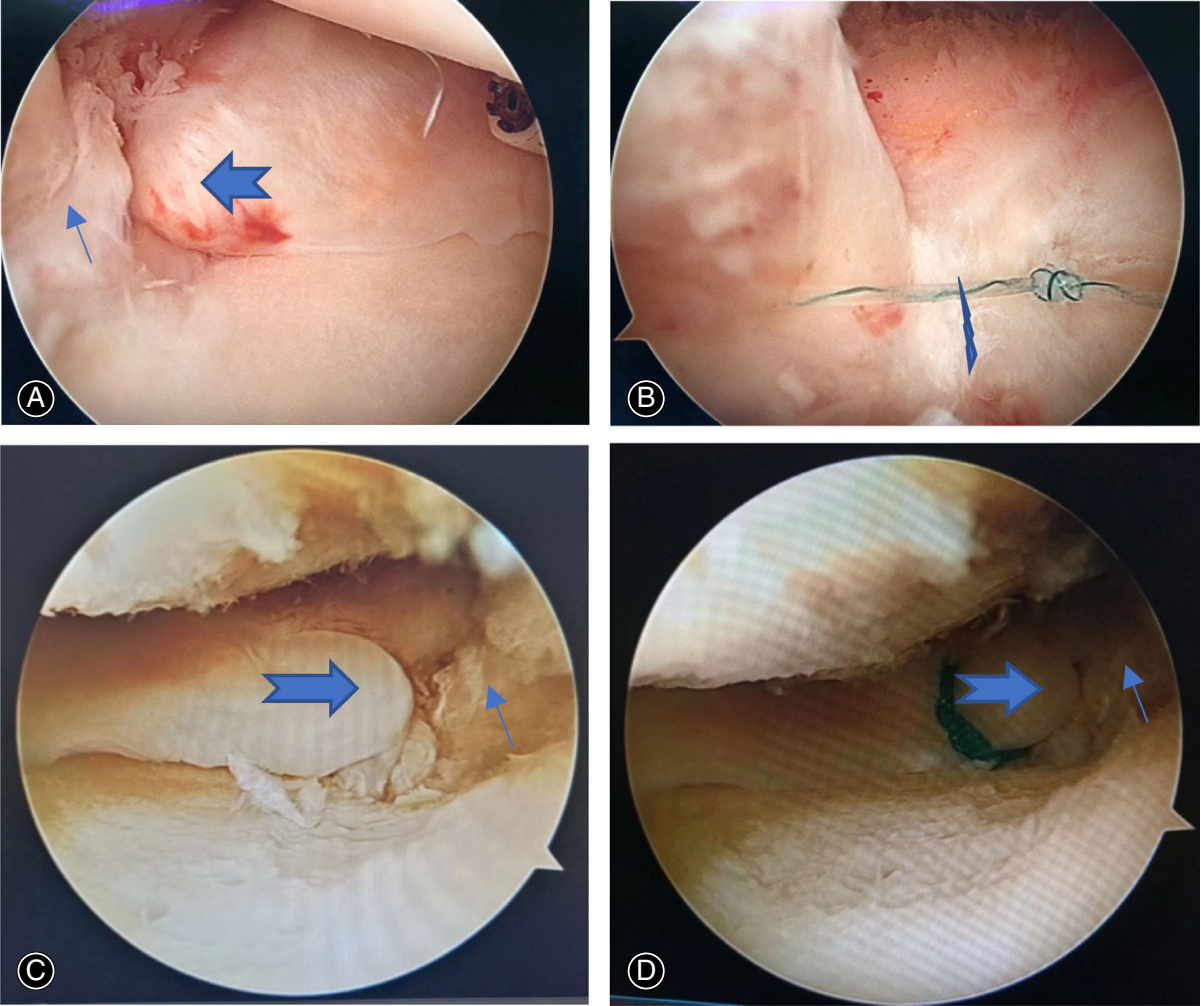
Arthroscopic all‐inside horizontal mattress suturing repair of MMPRT to PCL for a 67‐year‐old woman patient with right knee MMPRT and normal limb alignment. (A) A long arrow indicated the meniscus tissue remnants of medial meniscus posterior root attachment, a swallow‐tail arrow indicated the medial side meniscus tissue of MMPRT. (B) A lightening arrow indicated one arthroscopic all‐inside horizontal mattress suturing of medial side meniscus tissue of MMPRT to PCL, which also tied tissue remnants of medial meniscus posterior root attachment and medial side meniscus tissue of MMPRT together. Sometimes, another arthroscopic all‐inside horizontal mattress suturing could be added to increase suturing stability. (C) Another patient with left knee MMPRT, with same indication of a long arrow and a swallow‐tail arrow in Figure A. (D) After transtibial pullout repair, the tibial insertion of medial meniscus posterior root attachment was moved to a more medial location, while posterior root attachment remnants (long arrow) were left aside.

All arthroscopic surgeries were performed by the same attending surgeon. The patient was placed in supine position during operation under lumbar anesthesia. Tourniquet was applied to the base of the thigh. One supporting post was laterally attached to the operating table to hold the thigh in knee flexion. Standard anterolateral portal and anteromedial portal were used for routine arthroscopic examination and procedure. Routine arthroscopic examination was performed including patellofemoral joint and lateral compartment. The Type II MMPRT was confirmed with a probe examination through anteromedial portal. Cartilage of medial femoral condyle and medial tibial plateau was also evaluated. After debridement of medial tissue and root attachment remnants of posterior root tear with shaver, FAST‐FIX 360° meniscal repairing system (Smith & Nephew Endoscopy, Andover, MA) was used to perform all‐inside non‐anatomical suture fixation to PCL. Usually, one or two horizontal mattress sutures were made with first peek block insertion into PCL and then the second peek block into medial tissue of medial meniscus posterior root tear before suture knot tensioning (Figures 3 and 4). Then the suture knot was tightened to reduce medial side meniscus tissue of Type II MMPRT to PCL. The posterior root remnants and medial side meniscus tissue of Type II MMPRT were reduced and held together by suture knot tension. Not as medial meniscus body tear arthroscopic repairment, usually there was no need to perform pie‐crusting of medial collateral ligament to enlarge medial compartment space. Not as medial meniscus ramp lesion arthroscopic repairment, there is no need of intercondylar arthroscopic view and posteromedial portal establishment.

### Rehabilitation

After surgery, an ice bag was used on the front of kneecap to reduce knee effusion or edema for the first postoperative 2 days. When the anesthesia effect disappeared, ankle pumping was used to accelerate blood circulation of lower limb. On the first postoperative day, quadriceps strengthening was required through straight leg elevation exercise. Knee extension was performed with the help of ice bag (the first postoperative 2 days) or 1‐kg sand bag (after the first postoperative 2 days) on the front of kneecap. Knee flexion was performed through active exercise on bed without weight‐bearing. The range of motion was gradually increased to 90° at 3–4 weeks after surgery. The patients were allowed to walk with crutches and full extension with hinged knee brace on the first postoperative day. Partial weight‐bearing was allowed with toe‐touching. After postoperative 6 weeks, the brace was adjusted to 60° flexion to full extension during walking with crutches. After postoperative 2 months, the brace was adjusted to 90° flexion to full extension during walking with crutches. The range of motion of flexion was gradually increased to 120° on bed after postoperative 6 weeks. After postoperative 3 months, the patient was allowed to walk without crutches and knee brace. The full weight‐bearing was allowed after postoperative 3 months. Squatting was allowed after postoperative 4 months.

### Clinical and Radiological Evaluation

Preoperative MRI on operative knee was performed to diagnose MMPRT with cleft sign and ghost sign for all patients. Full‐length standing radiograph of lower limbs was also performed to confirm normal lower limb alignment (mechanical axis of lower limb is in one straight line through hip center, knee center, and ankle center). All patients were followed up at 3 months after surgery and final follow‐up. Satisfied clinical recovery was defined as no sudden posteromedial popping sensation and without posteromedial joint line tenderness with normal knee ROM and good quadriceps strength. At the final follow‐up, postoperative MRI was performed to confirm structural healing of MMPRT. The visual analog scale (VAS) score, the Lysholm score, and the Tegner activity score were recorded preoperatively and at the last follow up.

#### Visual Analog Scale (VAS) Score

The VAS score is a unidimensional measure of pain intensity in adult population. The score is determined by measuring the distance (cm) on the 10‐cm line (with 1 mm as one unit) between the “no pain” anchor and the patient's mark, providing a range of scores from 0 to 10. For pain intensity, the scale is most anchored by “no pain” (score of 0) and “pain as bad as it could be” or “worst imaginable pain” (score of 10). The following cut points for VAS have been recommended: no pain (0–0.4 cm), mild pain (0.5–4.4 cm), moderate pain (4.5–7.4 cm), and severe pain (7.5–10 cm).

#### The Lysholm Score

The Lysholm score is used to evaluate activities of daily living scales for patients with a variety of knee disorders including ligament and meniscus injuries, patellofemoral pain, etc. Eight factors are rated to produce an overall score on a point scale of 0 to 100. The factors of limp, support, and squatting are worth a potential of 5 points each; pain and instability 25 points each; swelling and stair climbing, 10 points each; and locking 15 points. Then an assignment is given as “excellent” for 95 to 100 points; “good” for 84 to 94 points, “fair” for 65 to 83 points, or “poor” for less than 65 points.

#### The Tegner Activity Score

The Tegner Activity Score aims to provide a standardized method in determining the level of activity prior to injury and level of activity post injury that can be documented on a numerical scale. The Tegner activity score is a one‐item score that graded activity based on work and sports activities on a scale of 0 to 10. Zero represents disability because of knee problems and 10 represents national‐ or international‐level soccer.

### Statistical Analysis

Statistical analyses were performed with SPSS version 21.0 (IBM Inc. Chicago, Illinois, United States). The Wilcoxon rank sum test was performed to determine the statistical significance between the preoperative results and those at the final follow up, to determine whether there was significant improvement in clinical outcome measures. *p* < 0.05 was considered statistically significant difference.

## Results

### Characteristics of Included Patients and Follow‐Up

Twenty patients (mean age 54.5 ± 19.5 years) were included in the present study. The demographic data were shown in Table 1. All 20 patients underwent complete imaging evaluation and clinical assessments preoperatively and at final follow‐up. The mean follow‐up duration was 32.5 ± 5.8 months.

**TABLE 1:** Preoperative demographic data

Demographics	Mean ± SD (range)
Age (years)	54.5 ± 19.5
Sex (male/female)	6/14
Operative side (right/left)	12/8
Follow‐up period (months)	32.5 ± 5.8
BMI (kg/m^2^)	23.5 ± 3.6
HKA angle (°)	0°–2° varus

### Clinical Outcomes

After 3 months, all patients manifested as no posteromedial pain and popping sensation in the knee. They walked without knee brace and crutches. After knee rehabilitation exercise, full range of motion can be achieved. However, deep squatting and stairs ascending/descending activity was allowed to prevent retear of medial meniscus posterior root until 3–4 months postoperatively.

### Functional Outcomes

The VAS score was significantly decreased from 6.5 ± 1.5 preoperatively to 2.1 ± 1.4 at the final follow‐up (*t* = 9.92, *P* < 0.01). The mean Lysholm score was significantly improved from 43.7 ± 10.9 preoperatively to 85.7 ± 10.8 (*t* = 72.35, *P* < 0.01). The median Tegner activity score was improved from 1.0 (range 1–4) to 3.0 (range 2–4) (*t* = 3.16, *P* < 0.01) (Table 2).

**TABLE 2:** Variation of functional results

Functional score	Preoperative score	Postoperative score	*t* value	*P* value
VAS score	6.5 ± 1.5	2.1 ± 1.4	9.92	< 0.01
Lysholm score	43.7 ± 10.9	85.7 ± 10.8	72.35	< 0.01
Tegner activity score	1.0	3.0	3.16	< 0.01

### Radiological Outcomes

**Fig. 5:**
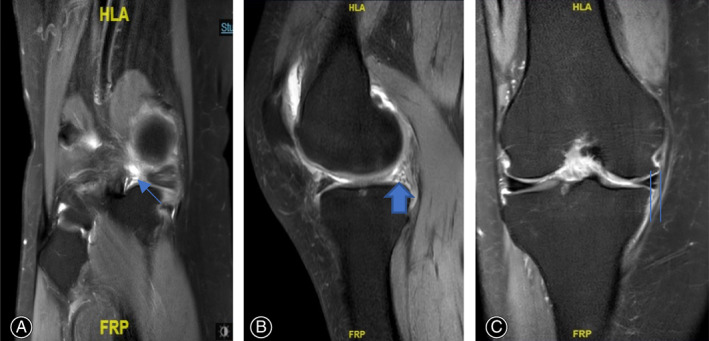
Thirty‐one months later after arthroscopic non‐anatomical repair of MMPRT to PCL of a 67‐year‐old woman patient with right knee MMPRT and normal limb alignment. Postoperative MRI images indicated almost complete healing of MMPRT. (A) MMPRT’s almost complete healing indicated by long fine arrow in T2 coronal view. (B) Almost triangular shape of MMPR indicated by long‐coarse arrow. (C) Decreased medial meniscus extrusion degree indicated by two lines.

At final follow‐up, on MRI, 12 cases (60%) had complete healing and eight cases (40%) had partial healing (Figure 5). The configuration of the repaired posterior root was almost normal, and the signal intensity of the preoperative defect site was high in T2 serials (not similar in signal to the normal meniscus) on sagittal, coronal, and axial images.

### Complication

For all 20 patients, there were no infection and neurovascular injury during follow‐up. The arthroscopic repair surgery is a safe procedure for MMPRT repair because of arthroscopic minimal invasion surgery and insertion of peek behind PCL and posteromedial capsule was far away from popliteal vessels and nerve.

## Discussion

Arthroscopic anatomical tibial pullout or anchor repair of MMPRT was a difficult arthroscopic technique to perform, especially suturing of medial side meniscus tissue of MMPR and avoiding damage of medial femoral condylar cartilage. The arthroscopic surgical procedure required special instruments for tibial tunnel drilling, anchor placement, and suture passing of medial side meniscus tissue of MMPR. The surgery was also time‐consuming and had a long learning curve for surgeons. On the other hand, posterior root attachment remnants were left aside during arthroscopic tibial pullout or anchor repair, which creates a new insertion of MMPR in more medial location of posterior part of medial tibial plateau than original attachment location. The medial side meniscus tissue would heal to more medial location of posterior part of medial tibial plateau which could increase tibiofemoral contact pressure^16^.

### Non‐Anatomic Arthroscopic All‐Inside Repair Technique

The horizontal mattress suturing between medial tissue of MMPRT and PCL can tie posterior root attachment remnants and medial meniscus tissue of MMPRT together which is useful for healing of both side medial meniscus tissue of MMPRT. Although there is space between MMPRT and PCL, the PCL provides a stabilizing structure for suturing of medial side meniscus tissue of MMPRT with remnants of posterior root attachments. However, it is not easy to perform direct horizontal mattress suturing between posterior root remnants tissue and medial side meniscus tissue of MMPRT, due to the small and unstable posterior root remnants tissue.

A biomechanical cadaver study reported that non‐anatomical suture fixation repair of MMPRT to PCL improved the contact area and resulted in pressures that were not significantly different from the intact state at most knee flexion angles^18^, which provided theory for our clinical practice for all‐inside arthroscopic horizontal mattress suturing of MMPRT to PCL. It is imperative to investigate whether PCL function may be influenced by the non‐anatomical repair or not. The small tissue of PCL was sutured and the sliding suture knot tension can be controlled to be not too much. So, the influence of PCL function will be lowered to a small degree.

It is relatively easy to perform arthroscopic horizontal suture mattress repair than tibial pullout or anchor suturing repair for MMPRT. The learning curve is relatively short. And the surgical procedure is not difficult to perform and can be performed rapidly. During surgery, there is also little risk to cause medial femoral condyle cartilage iatrogenic damage. The suture knot tension can be adjusted during surgery to hold the posterior root remnants and medial side meniscus tissue of Type II MMPRT together, which is useful for MMPRT healing.

Jiang *et al*.^19^ analyzed the risk factors of MMPRT suturing repair, with the greatest risk factor being more than 5° knee varus deformity. Knee varus can cause higher load in medial compartment. It was not easy for MMPRT repair to heal in such a mechanical circumstance. MMPRT repair was the treatment of choice for acute traumatic root tears without pre‐existing osteoarthritis and for chronic symptomatic root tears in relatively young patients who do not generally have osteoarthritis. If patients indicated for surgical treatment present with excessive varus malalignment (>5°), high tibial osteotomy (isolated or concomitant repair) may be attempted for a better outcome^20^. Thus, the patients with normal lower limb alignment were included in the present study.

### Short‐Term Clinical Outcome and Radiologic Healing Results

The clinical and radiological outcomes in follow‐up duration were satisfied. These patients recovered knee function without posteromedial popping sensation and pain and without knee squatting limitation. We mainly access the MMPRT healing through clinical symptom and sign, but not MRI image. Postoperative MRI showed complete structural healing or partial structural healing (Figure 5), although there was a high signal in MMPRT. Franky *et al*. reported that asymptomatic clinical menisci healing produces abnormal higher MRI signals even though they have stable unions, and that higher MRI signals at the site of repair represent edematous scar tissue, not true nonunion^21^. We believe that in symptom‐free patients, posterior root of medial meniscus is either histologically healed with PCL or posterior root remnants through scar tissue or acts as an autograft and fulfills the mechanical tasks of an injured meniscus.

In one‐word, non‐anatomical arthroscopic repair of MMPRT to PCL could yield beneficial outcomes in patients with normal lower limb alignment. It was an easy and reliable arthroscopic repair method for type II MMPRT.

### Reduction of Medial Meniscus Extrusion

In our study, we did not investigate the pullout degree of medial meniscus extrusion on MRI during follow‐up. The degree of medial meniscus extrusion could be reduced to a lower degree after MMPRT repair to PCL. The reason might be no more higher compression force in knee medial compartments. When the hoop tension was recovered after suturing, the medial meniscus extrusion will be reduced to some degree (Figure 5). From the perspective of medial meniscus extrusion, a landmark study pointed out that the amount of suture cutting‐out at the suture‐meniscus interface in tibial pullout suture repair might be a major suspect of displacement of repaired MMPRT^22^, ^23^. However, non‐anatomical arthroscopic repair of MMPRT to PCL might reduce meniscus extrusion to some degree because of stable suture with peek block in meniscus tissue, posteromedial capsule, or PCL. The peek block and suture behind the posteromedial capsule could produce some pullout force for meniscus extrusion. Furthermore, medial meniscus's function of shock absorption, load transmission, and knee stability were restored to almost normal after recovery of hoop tension according to Saltzman's cadaver biomechnical study^18^.

A modified repair method may be used to further reduce medial meniscus extrusion. For one thing, the horizontal mattress suturing can stabilize medial tissue of MMPRT and PCL. For another, a horizontal mattress suturing method with FAST‐FIX 360° underlying the tibial side of posterior horn of medial meniscus was performed to reduce posteromedial meniscal extrusion^24^. The remaining two sutures of the two horizontal mattress sutures were tied together to reduce medial meniscus extrusion.

## Conclusion

Non‐anatomical arthroscopic repair of MMPRT to PCL may yield beneficial clinical outcomes with higher rate of clinical healing in Type II MMPRT patients with normal lower limb malalignment. It is an easy and reliable alternative to the transtibial pullout suture or anchor suture repair.
